# Development and validation of a nomogram to predict mortality risk in patients with ischemic heart disease

**DOI:** 10.3389/fcvm.2023.1115463

**Published:** 2023-02-16

**Authors:** Long Yang, Xia Dong, Baiheremujiang Abuduaini, Nueraihemaiti Jiamali, Zulihuma Seyiti, Xue-Feng Shan, Xiao-Ming Gao

**Affiliations:** ^1^College of Pediatrics, Xinjiang Medical University, Ürümqi, China; ^2^Intensive Care Unit, Cardiovascular Center, First Affiliated Hospital of Xinjiang Medical University, Ürümqi, China; ^3^First Clinical Medical College, Xinjiang Medical University, Ürümqi, China; ^4^Pediatric Cardiothoracic Surgery, First Affiliated Hospital of Xinjiang Medical University, Ürümqi, China; ^5^Department of Cardiology, State Key Laboratory of Pathogenesis, Prevention and Treatment of High Incidence Diseases in Central Asia, First Affiliated Hospital of Xinjiang Medical University, Ürümqi, China; ^6^Xinjiang Key Laboratory of Medical Animal Model Research, Ürümqi, China; ^7^Clinical Medical Research Institute, Xinjiang Medical University, Ürümqi, China

**Keywords:** nomogram, ischemic heart disease, LASSO, mortality, machine learning

## Abstract

**Background:**

Ischemic Heart Disease (IHD) is the leading cause of death from cardiovascular disease. Currently, most studies have focused on factors influencing IDH or mortality risk, while few predictive models have been used for mortality risk in IHD patients. In this study, we constructed an effective nomogram prediction model to predict the risk of death in IHD patients by machine learning.

**Methods:**

We conducted a retrospective study of 1,663 patients with IHD. The data were divided into training and validation sets in a 3:1 ratio. The least absolute shrinkage and selection operator (LASSO) regression method was used to screen the variables to test the accuracy of the risk prediction model. Data from the training and validation sets were used to calculate receiver operating characteristic (ROC) curves, C-index, calibration plots, and dynamic component analysis (DCA), respectively.

**Results:**

Using LASSO regression, we selected six representative features, age, uric acid, serum total bilirubin, albumin, alkaline phosphatase, and left ventricular ejection fraction, from 31 variables to predict the risk of death at 1, 3, and 5 years in patients with IHD, and constructed the nomogram model. In the reliability of the validated model, the C-index at 1, 3, and 5 years was 0.705 (0.658–0.751), 0.705 (0.671–0.739), and 0.694 (0.656–0.733) for the training set, respectively; the C-index at 1, 3, and 5 years based on the validation set was 0.720 (0.654–0.786), 0.708 (0.650–0.765), and 0.683 (0.613–0.754), respectively. Both the calibration plot and the DCA curve are well-behaved.

**Conclusion:**

Age, uric acid, total serum bilirubin, serum albumin, alkaline phosphatase, and left ventricular ejection fraction were significantly associated with the risk of death in patients with IHD. We constructed a simple nomogram model to predict the risk of death at 1, 3, and 5 years for patients with IHD. Clinicians can use this simple model to assess the prognosis of patients at the time of admission to make better clinical decisions in tertiary prevention of the disease.

## 1. Introduction

In many countries, cardiovascular disease is increasing annually and poses a serious threat to human life (1). Ischemic Heart Disease (IHD) is the leading cause of death from cardiovascular disease (2). IHD is caused by plaque accumulation along the inner wall of the coronary artery, resulting in partial or complete occlusion of the coronary artery, thereby reducing blood flow to the heart (3). Long-term IHD imbalances coronary blood flow regulatory mechanisms, including ion channels, leading to the development of hypoxia, fibrosis, and tissue death, which may determine the loss of myocardial function (4). These mechanisms will produce serious complications such as heart failure, ventricular arrhythmias, and myocardial infarction, ultimately leading to the death of patients (5). IHD mortality remains persistently elevated in most developing countries worldwide, placing a significant economic and resource burden on health and public health systems (6). Therefore, early identification of the risk of mortality in IHD patients becomes particularly important.

Numerous factors influence the risk of mortality in patients with IHD, including gender, age, blood pressure, smoking, alcohol consumption, and the presence of comorbidities, such as a history of diabetes and hypertension (7). Besides, the predictive role of leukocytes, neutrophils, lymphocytes, monocytes, eosinophils, basophils, erythrocytes, hemoglobin, and platelets for cardiovascular disease has also been reported (8). Furthermore, there are several recognized risk factors for cardiovascular disease, such as creatinine, uric acid, fasting blood glucose, high-density lipoprotein cholesterol, low-density lipoprotein cholesterol, total bilirubin, albumin, alanine aminotransferase, AST, lactate dehydrogenase, γ-glutamyl transferase, alkaline phosphatase, and creatine kinase, all of which play an important role in the progression of cardiovascular disease (9–12). In addition, the left ventricular ejection fraction is used to assess important echocardiographic parameters of cardiac function in IHD patients (13).

Most of the current research has focused on specific risk factors affecting death in IDH, and few predictive models have been used for the risk of death in patients with IHD. In this study, we constructed an effective nomogram prediction model to predict the risk of death in IHD patients by machine learning. The nomogram can be used to accurately predict the risk of disease or complications in many cases (14), and our study is based on data from the largest medical center in Xinjiang, China, to more comprehensively consider the impact of various factors on prognosis to develop and validate a simple, practical, and accurate risk prediction tool for IHD patients. The nomograms we developed can be used to assess the risk of death at 1, 3, and 5 years in patients with IHD, providing clinicians with a way to identify people at high risk of death from IDH at admission, and then appropriate interventions to prolong the survival time of patients.

## 2. Materials and methods

### 2.1. Participants

We selected the data of patients who attended the First Affiliated Hospital of Xinjiang Medical University from 2010 to 2017, and a total of 2,043 patients with ischemic heart disease were included in this study. According to the Declaration of Helsinki, informed consent was obtained and the patients’ identities were completely concealed before including the data in the study. The inclusion and exclusion criteria were as follows.

#### 2.1.1. Inclusion criteria

Diagnosis on admission was ischemic heart disease: (1) angina pectoris or equivalent symptoms occurring during exercise, rest, or nitroglycerin relief. (2) Electrocardiogram showed significant myocardial ischemia type, and an exercise test revealed myocardial ischemia. (3) Coronary angiography showed that the degree of coronary artery stenosis was greater than 50%.

#### 2.1.2. Exclusion criteria

(1) Congenital heart disease. (2) Severe heart valve disease (mitral stenosis, mitral regurgitation, aortic stenosis, and aortic insufficiency); (3) Pericardial disease (acute pericarditis, constrictive pericarditis). (4) Heart failure caused by non-cardiac factors (severe infection, anemia); (5) Thyroid dysfunction. (6) Severe liver damage (alanine aminotransferase and aspartate aminotransferase three times higher than the upper limit of normal); (7) Severe renal impairment. (8) Malignant tumor.

**FIGURE 1:**
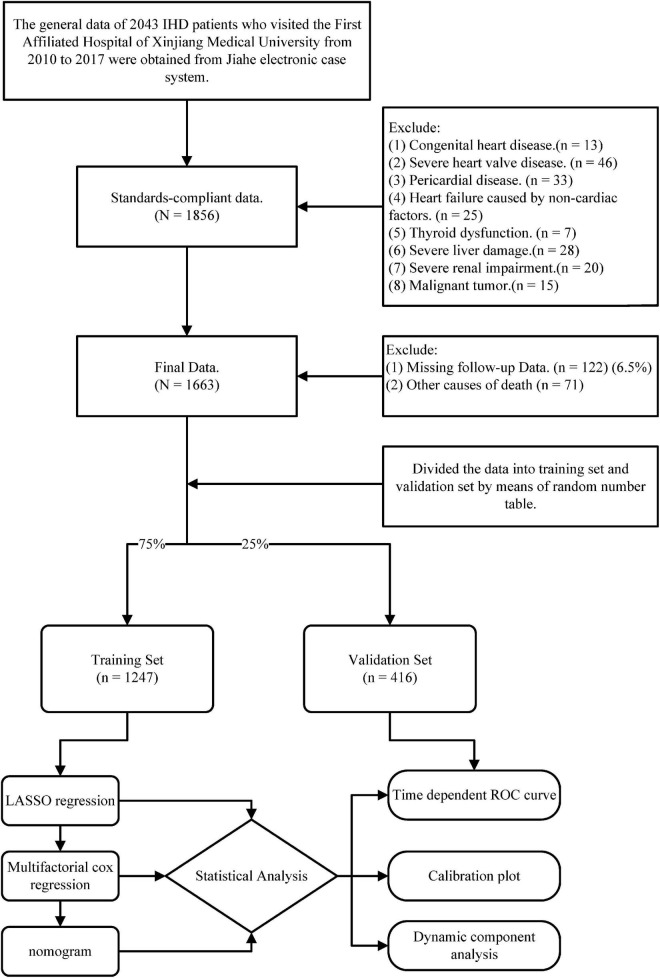
The flow chart presents the entire process of patient follow-up, data collection, and statistical analysis in this study.

We followed the patients for 5 years with the endpoint event of cardiovascular death. After excluding samples with missing follow-up and other causes of death, we finally included a sample of 1,663 cases (Figure 1).

### 2.2. Data source

Patient clinical data was derived from information stored in the Jiahe electronic case system at the time of initial presentation. Include gender, smoking history, alcohol history, diabetes history, hypertension history, age, systolic blood pressure, diastolic blood pressure, and other general information. All patients were asked to fast on the next day and 8 mL of venous blood was collected at admission, and routine blood data were measured using an automatic hematology analyzer XN-1000 (Sysmex, Japan). Biochemical data such as serum creatinine, uric acid, and fasting blood glucose were measured using an automatic biochemical analyzer DXC800 (Beckman Coulter, USA). Echocardiography was performed within 24 h after admission using a VIVID7 color Doppler ultrasound diagnostic apparatus (GE, USA) to measure left ventricular ejection fraction.

### 2.3. Statistical analysis

Statistical analysis of data from this study was performed using R software (The R Foundation).^1^ Two-sided *P* < 0.05 was considered statistically significant in all analyses. First, the patients were divided into training and validation sets for external validation with R software satisfying a 3:1 ratio. A normality test was performed on both data sets, and the study data were found to be non-normally distributed. Continuous variables are therefore presented as medians (quartiles) and categorical variables as frequencies (percentages). The Mann–Whitney *U*-test is used for all continuous variables and the Chi-squared test is used for all categorical variables. There were no statistically significant differences between the two groups in the above tests (*P* > 0.05). Data from the training set were then analyzed using least absolute shrinkage and selection operator (LASSO) regression. Variables with non-zero coefficients in the LASSO regression model were used to build nomogram prediction models. Multivariate Cox regression was used to verify whether the variables in the model were statistically significant. To test the accuracy of the risk prediction model, receiver operating characteristic (ROC) curves, C-Index, calibration plot, and dynamic component analysis (DCA) were calculated using data from the training and validation sets, respectively.

## 3. Results

### 3.1. Baseline characteristics of participants

Ultimately, 1,663 participants with IHD were enrolled in the study. During a median follow-up of 42 months, 693 patients died. The median age of the patients was 47 years, with 1,264 (76%) males and 399 (24%) females. For external validation, the total number of cases was divided into a training set (1,247 patients) and a validation set (416 patients) in a ratio of approximately 3:1. The median age of the patients in the training set was 74 years, with 950 males and 297 females. During a median follow-up period of 42 months, 511 patients died. The median age of the patients in the validation set was 73 years, with 314 males and 102 females. During a median follow-up period of 42 months, 182 patients died. The two groups had no statistically significant difference in general information or laboratory test data (Table 1).

**TABLE 1:** Characteristics of the participants in different groups.

Characteristic	Overall (*n* = 1,663)	Training set (*n* = 1,247)	Validation set (*n* = 416)	*P*-value
Gender				0.823
Male	1264 (76.0)	950 (76.2)	314 (75.5)	
Female	399 (24.0)	297 (23.8)	102 (24.5)	
Smoking history	731 (44.0)	537 (43.1)	194 (46.6)	0.225
Drinking history	463 (27.8)	343 (27.5)	120 (28.8)	0.642
Hypertension history	1009 (60.7)	763 (61.2)	246 (59.1)	0.494
Diabetes history	708 (42.6)	525 (42.1)	183 (44)	0.537
Age, years	67 (57, 75)	68 (57, 75)	67 (57, 75)	0.889
SBP, mmHg	122 (110, 138)	121 (110, 137)	123.5 (110, 140)	0.309
DBP, mmHg	73 (65, 80)	74 (65, 80)	73 (65, 80)	0.634
WBC, 10^9^/L	6.88 (5.73, 8.225)	6.83 (5.74, 8.17)	7.02 (5.73, 8.48)	0.250
Neutrophil, 10^9^/L	4.36 (3.45, 5.56)	4.35 (3.45, 5.53)	4.38 (3.48, 5.63)	0.536
Lymphocyte, 10^9^/L	1.62 (1.24, 2.05)	1.60 (1.23, 2.05)	1.67 (1.27, 2.04)	0.406
Monocyte, 10^9^/L	0.55 (0.43, 0.69)	0.54 (0.43, 0.68)	0.56 (0.45, 0.71)	0.023
Eosinophils, 10^9^/L	0.12 (0.07, 0.20)	0.12 (0.07, 0.20)	0.13 (0.07, 0.20)	0.854
Basophils,10^9^/L	0.03 (0.02, 0.04)	0.03 (0.01, 0.04)	0.03 (0.02, 0.04)	0.196
RBC,10^9^/L	4.49 (4.04, 4.87)	4.49 (4.04, 4.86)	4.49 (4.05, 4.97)	0.218
Hemoglobin, g/L	135 (123, 147)	135 (122.5, 147)	136 (125, 148)	0.171
PLT, 10^9^/L	201 (162, 245)	201 (164, 246)	199 (157.75, 243)	0.177
Cr, umol/L	85 (70, 105)	85 (70, 104)	85.44 (70, 108)	0.316
UA, umol/L	378 (309.65, 466.505)	377 (309.65, 459.75)	381.5 (309.46, 482.31)	0.335
Glu, mmol/L	6.1 (4.92, 8.43)	6.04 (4.89, 8.29)	6.46 (5.03, 8.70)	0.067
HDL-C, mmol/L	0.92 (0.76, 1.11)	0.92 (0.77, 1.10)	0.92 (0.75, 1.12)	0.765
LDL-C, mmol/L	2.13 (1.65, 2.73)	2.13 (1.65, 2.71)	2.16 (1.65, 2.77)	0.823
TBil, umol/L	13.78 (9.90, 19.48)	13.7 (9.70, 19.3)	14.09 (10.33, 20.01)	0.091
Albumin, g/L	38.1 (34.5, 41.1)	38.0 (34.3, 40.9)	38.4 (34.5, 41.7)	0.086
ALT, U/L	20.7 (16.3, 28.9)	20.5 (16.3, 28.4)	21.1 (16.4, 31.0)	0.330
AST, U/L	20.67 (14.17, 32.365)	20.72 (14, 31.65)	20.55 (14.6, 34.69)	0.348
LDH, U/L	190 (160.105, 232.24)	190 (160.1, 231.2)	190.09 (161.56, 237)	0.480
GGT, U/L	32.0 (20.8, 54.0)	31.5 (20.5, 52.7)	32.0 (21.4, 56.0)	0.183
ALP, U/L	72.9 (59.0, 90.5)	73.0 (59.0, 90.3)	72.0 (59.0, 90.56)	0.883
CK, U/L	69.9 (47.3, 102.1)	68.6 (47.0, 100.8)	72.2 (50.0, 110.3)	0.074
LVEF, %	42 (37, 48)	42 (37, 48)	42 (36, 48)	0.375

Data are expressed as medians with interquartile ranges or percentage. SBP, systolic blood pressure; DBP, diastolic blood pressure; WBC, white blood cell count; PLT, blood platelet count; RBC, red blood cell count; Cr, creatinine; UA, uric acid; Glu, glucose; TBil, total bilirubin; LDL, low-density lipoprotein cholesterol; HDL, high-density lipoprotein cholesterol; AST, aspartate aminotransferase; ALT, alanine transaminase; LDH, lactic dehydrogenase; GGT, glutamyl transferase; ALP, alkaline phosphatase; CK, creatine kinase; LVEF, left ventricular ejection fraction.

### 3.2. Construction of clinical prediction models

**FIGURE 2:**
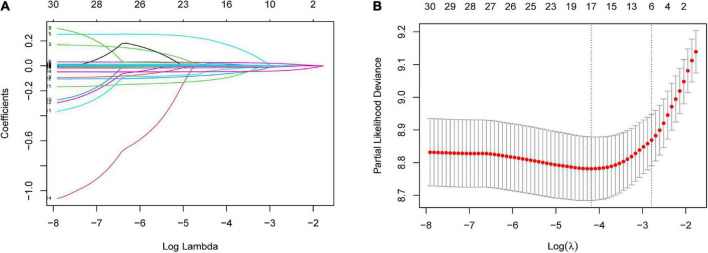
Risk factors contributing to death within 5 years in patients with IHD were selected using LASSO regression models. **(A)** LASSO coefficient profiles of the 31 features. A coefficient profile plot was generated against the log(lambda) sequence. **(B)** Optimal parameter (lambda) selection in the LASSO model used five-fold cross-validation based on minimum criteria. The partial likelihood deviance (binomial deviance) curve was plotted vs. log(lambda). Dotted vertical lines were drawn at the optimal values by using the minimum criteria and the 1 SE of the minimum criteria (the 1-SE criteria). LASSO coefficient profiles of the six features.

**FIGURE 3:**
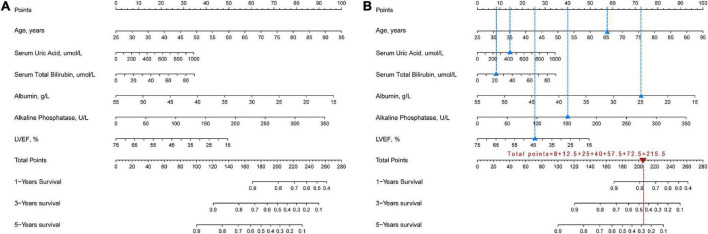
Nomogram for assessing the risk of death within 1, 3, 5 years in IHD patients. **(A)** Complete nomogram. **(B)** How to use the nomogram. Blue triangles show clinical indicators for a hypothetical IHD patient and blue dashed lines show the corresponding scores. Red triangles show the total scores and the red dashed lines show the corresponding survival rates.

Least absolute shrinkage and selection operator regression analysis was performed on the training set data. By selecting non-zero features in the LASSO regression analysis results, the number of potential predictor variables was reduced from 18 to 6 (Figures 2A, B). Including age, uric acid, serum total bilirubin, albumin, alkaline phosphatase, and left ventricular ejection fraction. Multivariate Cox regression analysis was then used to verify whether each variable was statistically significant (Table 2). Finally, the above six risk profile factors were used to develop a predictive model to predict the risk of death at 1, 3, and 5 years in IHD patients and displayed in the form of nomograms (Figure 3).

**TABLE 2:** Based on the coefficients and Lambda.1se values of the LASSO regression of the training set, multifactorial COX regression to validate the validity of each variable.

Characteristic	LASSO regression		Multifactorial COX regression		
	Coefficients	Lambda.1se	HR	95% CI	*P*-value
Age	0.018116	0.06137	1.034	(1.034–1.025)	<0.001
UA	0.000054		1.001	(1.001–1.000)	0.023
TBil	0.000141		1.009	(1.009–1.001)	0.032
Albumin	−0.039768		0.945	(0.945–0.930)	<0.001
ALP	0.002561		1.006	(1.006–1.004)	<0.001
LVEF	−0.008666		0.981	(0.981–0.972)	<0.001

Coefficients, coefficients of each variable in LASSO regression; Lambda.1se, among all lambda values, the lambda value of the simplest model within a variance of the mean value of the minimum target parameter is obtained; HR, hazard ratios from multifactorial COX regressions performed to verify the validity of each variable; 95% CI, 95% confidence interval of the hazard ratio; UA, uric acid; TBil, total bilirubin; ALP, alkaline phosphatase; LVEF, left ventricular ejection fraction.

### 3.3. Validation of clinical prediction models

**FIGURE 4:**
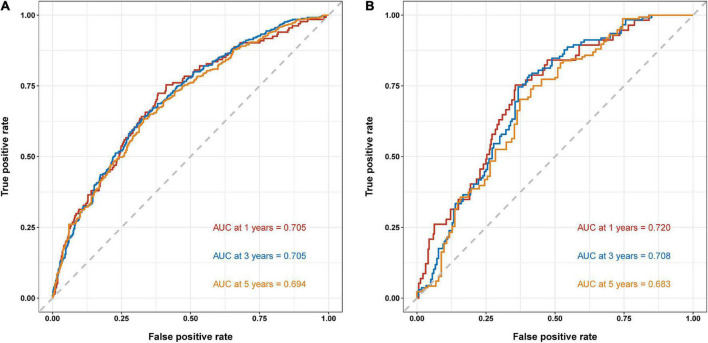
Time-dependent receiver operating curve (ROC) analysis of nomogram model. **(A)** Training set. **(B)** Validation set.

**FIGURE 5:**
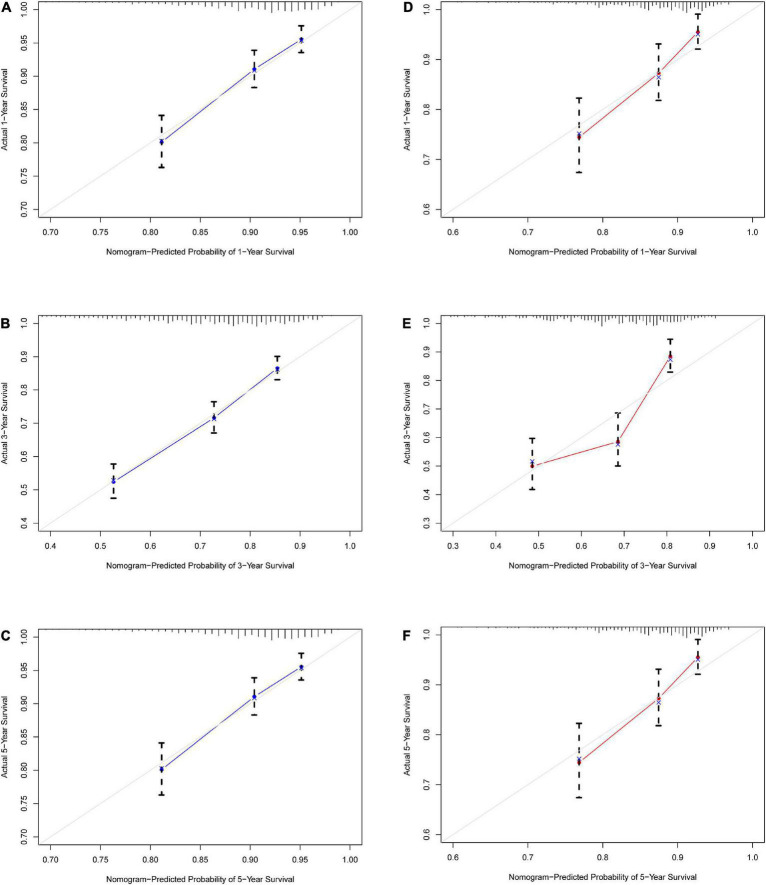
Calibration curve for IHD 1-, 3-, and 5-year mortality risk prediction in the array. **(A)** Training set 1 year. **(B)** Training set 3 years. **(C)** Training set 5 years. **(D)** Validation set 1 year. **(E)** Validation set 3 years. **(F)** Validation set 5 years. The x-axis represents the predicted incidence risk. The y-axis represents the actual diagnosed CHD. The diagonal dotted line represents a perfect prediction by an ideal model. The solid line represents the performance of the nomogram; a closer fit to the diagonal dotted line represents a better prediction.

**FIGURE 6:**
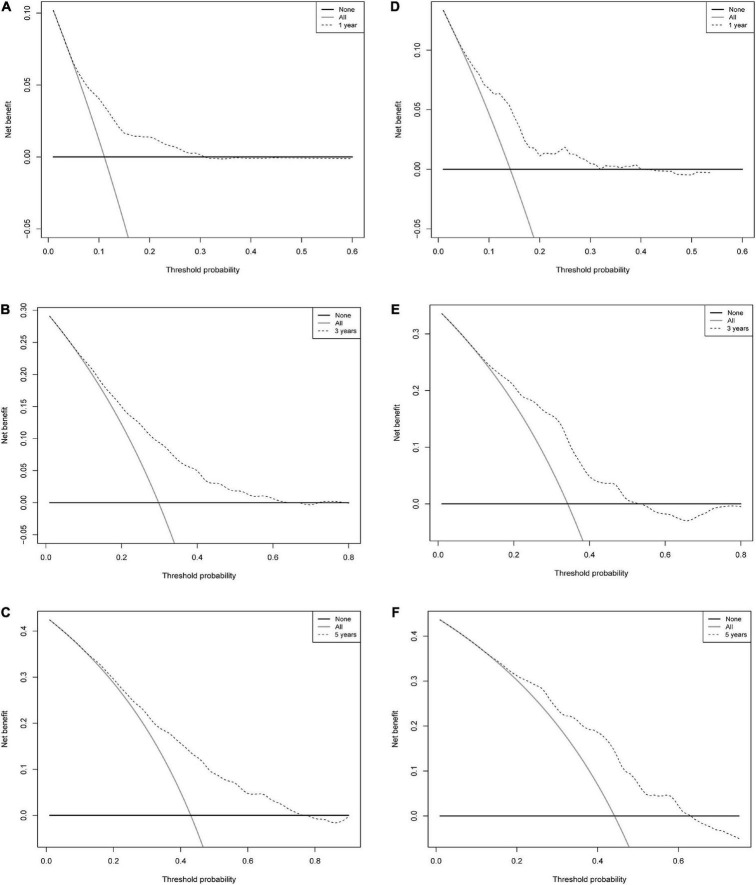
Decision curve analysis of a nomogram of mortality risk at 1, 3, and 5 years in patients with IHD. **(A)** Training set 1 year. **(B)** Training set 3 years. **(C)** Training set 5 years. **(D)** Validation set 1 year. **(E)** Validation set 3 years. **(F)** Validation set 5 years. The y-axis represents the net benefit. Dashed lines represent the IHD mortality risk nomogram. The thin solid line represents the hypothesis that all patients died. Thin solid lines represent the hypothesis of no patient death.

To validate the reliability of the prediction model, the model is tested using data from the training and validation sets. As assessed by time-dependent receiver operating curve (ROC) analysis, the time-dependent accuracy of the nomogram model in predicting mortality risk at 1, 3, and 5 years is shown (Figure 4). C-index based on the training set 1, 3, and 5 years were 0.705 (0.658–0.751), 0.705 (0.671–0.739), and 0.694 (0.656–0.733), respectively; C-index based on the validation set 1, 3, and 5 years were 0.720 (0.654–0.786), 0.708 (0.650–0.765), and 0.683 (0.613–0.754), respectively (Table 3). In addition, to further validate the model, we analyzed the data from the training and validation sets using calibration plots (Figure 5) and decision curve analysis (Figure 6), and the results show that both calibration plots and DCA curves perform well.

**TABLE 3:** Time-dependent receiver operating curves (ROC) for training and validation sets.

	Training set		Validation set	
Time	C-Index	95% CI	C-Index	95% CI
1 year	0.705	(0.658–0.751)	0.720	(0.654–0.786)
3 years	0.705	(0.671–0.739)	0.708	(0.650–0.765)
5 years	0.694	(0.656–0.733)	0.683	(0.613–0.754)

## 4. Discussion

In this study, variables associated with mortality risk in IHD patients were screened by LASSO regression, a method that penalizes complex models with regularized parameters and identifies parameters that minimize mean square error, penalizing insignificant coefficients to zero (15). Finally, we screened six variables, age, uric acid, serum total bilirubin, albumin, alkaline phosphatase, and left ventricular ejection fraction, which had a significant impact on the risk of death in IHD patients, and then we constructed a nomogram prediction model, which is a two-dimensional graph, which uses approximate graph calculation of mathematical functions, is friendly in interface, more accurate, and has easily understood results and has been widely used in the medical field (16).

Our study showed that if an IHD patient was 65 years old, UA was 400 mmol/L, TBil was 20 mmol/L, albumin was 25 mmol/L, ALP was 150 U/L, and LVEF was 45%, the 1-, 3-, and 5-year survival rates of this patient were approximately 78, 45, and 29%, respectively (Figure 3B).

Through LASSO regression and Cox regression analysis, we concluded that the characteristics influencing the risk of death in IHD patients based on the training group in this study included age, uric acid, serum total bilirubin, albumin, alkaline phosphatase, and left ventricular ejection fraction, and the nomograms showed moderately good predictive ability by time-dependent ROC curve analysis, C-index, calibration plot, and DCA curve. Therefore, the impact of six factors cannot be ignored, and they are all strongly associated with the risk of mortality in IHD patients.

Our study suggests that age is one of the most important contributing factors to mortality risk in IHD patients. It has been shown that there is an exponential rise in mortality with increasing age in patients with IHD in both developed and developing countries (6). Increasing age is a major cause of vascular endothelial dysfunction, and aging endothelial cells accelerate the course of cardiovascular disease (17, 18). The mechanism may be that aging decreases circulating CD31 + T cell numbers and migration capacity, which leads to increased susceptibility to endothelial cell apoptosis, shortened telomere length, and decreased telomerase activity (19). In addition, aging leads to reduced cardiomyocyte turnover, increased levels of reactive oxygen species, DNA damage, organelle dysfunction, and accumulation of oxidized proteins and lipids, which leads to decreased cell quality control and accelerates the aging of the heart (20–22).

Uric acid is the end product of purine metabolism, and most scholars believe that the increase of uric acid will significantly increase the risk of cardiovascular disease, promote cardiovascular damage, and increase cardiovascular morbidity and mortality (23). In this study, uric acid was a risk factor for mortality in IHD patients. It has been shown that long-term hyperuricemia may lead to increased vascular sodium deposition and associated inflammatory burden (24). In addition, uric acid can lead to increased autophagy and cardiac hypertrophy in cardiomyocytes by increasing AMPK-ULK1 signaling pathway activity (25).

Previous studies have shown that mild increases in bilirubin protect against cardiovascular morbidity and mortality (26). Nevertheless, our study found that increased serum total bilirubin levels increased the risk of death in patients with IHD. However, several meta-analyzes have shown an L- or U-shaped association between serum bilirubin and the prognosis of coronary artery disease (27–29). In this study, the median bilirubin concentration in patients was 13.78 (9.90, 19.48), and one study demonstrated that the protective effect of bilirubin in patients with coronary heart disease increased with increasing bilirubin levels when bilirubin levels ranged from 3.42 to 13 mmol/L. When the bilirubin level exceeded 13 mmol/L, the protective effect of bilirubin was weakened, and the risk effect gradually emerged as the bilirubin level further increased (30). There is no specific mechanism to explain this phenomenon, but some studies suggest that stress-induced increases in heme oxygenase-1 activity may be exacerbating the process of coronary artery disease (29).

Serum albumin accounts for about 50% of the total plasma protein concentration and is one of the most abundant circulating proteins in the human body and an important indicator of nutritional status (31). Our study suggests an association between albumin and the risk of death in patients with IHD. Albumin has many physiological properties, including anti-inflammatory, antioxidant, and antiplatelet aggregation activities, and it also plays an important role in fluid exchange through capillary membranes (32, 33). Maintaining normal albumin levels can reduce the risk of death from ischemic heart disease (34). Low albumin levels decrease intravascular colloid osmotic pressure and increase inflammation and infectivity (35). This may accelerate the process of vascular atherosclerosis in patients with IHD, which in turn leads to serious complications such as myocardial infarction and heart failure (36, 37).

In this study, ALP was a risk factor for mortality risk in IHD patients. ALP is an enzyme responsible for hydrolyzing phosphates and releasing inorganic phosphates. Several studies have shown a significant linear association between ALP and the development of cardiovascular disease and death (38, 39). The mechanism may be that high serum phosphate levels lead to extracellular matrix degradation, osteochondrocyte changes, and increased production of reactive oxygen species, and stimulate osteoblast transcriptional programs in the vascular system in vascular smooth muscle (40). Calcification of vascular smooth muscle may therefore establish an association between ALP and the risk of death in IHD patients (41).

Left ventricular ejection fraction is the ratio of ventricular volume ejected during systole (stroke volume) to ventricular blood volume at the end of diastole (end-diastolic volume) and is widely used by clinicians as an important indicator for assessing cardiac function (42). Our study showed that LVEF was significantly associated with IHD mortality risk. LVEF is a powerful predictor of serious cardiovascular diseases such as heart failure, myocardial infarction, and arrhythmia (43, 44). In addition, it has been shown that ischemic heart disease patients with severe LVEF reduction will have a significantly increased risk of death (45).

Overall, we created a nomogram model to predict the 1-, 3-, and 5-year risk of death in patients with IHD, which helps clinicians understand the important risk factors affecting the development of death at the time of admission in patients with IHD, thereby taking reasonable tertiary preventive measures, which in turn reduces mortality and improves patient survival. In addition, the six indicators in the model are economical, non-invasive, simple, easily available, and the operation is not limited by hospital conditions.

## 5. Advantages and limitations

The key strength of this study is that machine learning was used to screen out several important factors affecting the risk of death in a wide range of indicators, and these parameters were relatively easy to obtain. However, there are still some limitations in this study, first, this study is a retrospective study and there may be unknown confounding factors. Second, this study only collected the data of patients at admission and did not evaluate the way patients were treated for the disease such as through medication and surgery. Finally, this study is a single-center cohort evaluation, and multi-center, large-sample studies are still needed to optimize and validate the model in the future.

## 6. Conclusion

This study showed that age, uric acid, serum total bilirubin, albumin, alkaline phosphatase, and left ventricular ejection fraction were significantly associated with the risk of death in patients with IHD, and we constructed a simple nomogram model to predict the risk of death in patients with IHD at 1, 3, and 5 years, and clinicians could assess the prognosis of patients through this simple model at the time of patient admission to make better clinical decisions in the tertiary prevention of the disease.

## Data availability statement

The original contributions presented in this study are included in this article/supplementary material, further inquiries can be directed to the corresponding authors.

## Ethics statement

The studies involving human participants were reviewed and approved by Medical Ethics Committee of the First Affiliated Hospital of Xinjiang Medical University. The patients/participants provided their written informed consent to participate in this study.

## Author contributions

LY and XD contributed to the conception and design, acquisition, drafting of the manuscript, and critical revision for important intellectual content. BA, NJ, and ZS contributed to the interpretation of the data and analysis. X-FS and X-MG contributed to the conception and design, reviewing of the manuscript, and critical revision for important intellectual content. All authors approved the final version and agreed to be accountable for all aspects of the work.

## Footnotes

1 http://www.r-project.org; version 4.2.1.
